# Влияние ИМТ на острый период COVID-19 и риски, формирующиеся в течение года после выписки. Находки субанализа регистров АКТИВ и АКТИВ 2

**DOI:** 10.14341/probl13165

**Published:** 2022-12-20

**Authors:** А. Г. Арутюнов, Е. И. Тарловская, Г. Р. Галстян, Т. И. Батлук, Р. А. Башкинов, Г. П. Арутюнов, Ю. Н. Беленков, А. О. Конради, Ю. М. Лопатин, А. П. Ребров, С. Н. Терещенко, А. И. Чесникова, Г. Г. Айрапетян, А. П. Бабин, И. Г. Бакулин, Н. В Бакулина, Л. А. Балыкова, А. С. Благонравова, М. В. Болдина, М. И. Бутомо, А. Р. Вайсберг, А. С. Галявич, В. В. Гомонова, Н. Ю. Григорьева, И. В. Губарева, И. В. Демко, А. В. Евзерихина, А. В. Жарков, А. А. Затейщикова, У. К. Камилова, З. Ф. Ким, Т. Ю. Кузнецова, А. Н. Куликов, Н. В. Ларева, Е. В. Макарова, С. В. Мальчикова, С. В. Недогода, М. М. Петрова, И. Г. Починка, К. В. Протасов, Д. Н. Проценко, Д. Ю. Рузанов, С. А. Сайганов, А. Ш. Сарыбаев, Н. М. Селезнева, А. Б. Сугралиев, И. В. Фомин, О. В. Хлынова, О. Ю. Чижова, И. И. Шапошник, Д. А. Щукарев, А. К. Абдрахманова, С. А. Аветисян, О. Г. Авоян, К. К. Азарян, Г. Т. Аймаханова, Д. А. Айыпова, А. Ч. Акунов, М. К. Алиева, А. Р. Алмухамбедова, А. В. Апаркина, О. Р. Арусланова, Е. Ю. Ашина, О. Ю. Бадина, О. Ю. Барышева, А. С. Батчаева, А. М. Битиева, И. У. Бихтеев, Н. А. Бородулина, М. В. Брагин, В. А. Бражник, А. М. Буду, Г. А. Быкова, К. Р. Вагапова, Д. Д. Варламова, Н. Н. Везикова, Е. А. Вербицкая, О. Е. Вилкова, Е. А. Винникова, В. В. Вустина, Е. А. Галова, В. В. Генкель, Д. Б. Гиллер, Е. И. Горшенина, Е. В. Григорьева, Е. Ю. Губарева, Г. М. Дабылова, А. И. Демченко, О. Ю. Долгих, М. Ы. Дуйшобаев, Д. С. Евдокимов, К. Е. Егорова, А. Н. Ермилова, А. Е. Желдыбаева, Н. В. Заречнова, Ю. Д. Зимина, С. Ю. Иванова, Е. Ю. Иванченко, М. В. Ильина, М. В. Казаковцева, Е. В. Казымова, Ю. С. Калинина, Н. А. Камардина, А. М. Караченова, И. А. Каретников, Н. А. Кароли, М. Х. Карсиев, Д. С. Каскаева, К. Ф. Касымова, Ж. Б. Керимбекова, Е. С. Ким, Н. В. Киселева, Д. А. Клименко, А. В. Климова, О. В. Ковалишена, С. В. Козлов, Е. В. Колмакова, Т. П. Колчинская, М. И. Колядич, О. В. Кондрякова, М. П. Коновал, Д. Ю. Константинов, Е. А. Константинова, В. А. Кордюкова, Е. В. Королева, А. Ю. Крапошина, Т. В. Крюкова, А. С. Кузнецова, Т. Ю. Кузьмина, К. В. Кузьмичев, Ч. К. Кулчороева, Т. В. Куприна, И. М. Куранова, Л. В. Куренкова, Н. Ю. Курчугина, Н. А. Кушубакова, В. И. Леванкова, А. А. Ледяева, Т. В. Лисун, В. Е. Лисянская, Н. А. Любавина, Н. А. Магдеева, К. В. Мазалов, В. И. Майсеенко, А. С. Макарова, А. М. Марипов, Н. В. Марков, А. А. Марусина, Е. С. Мельников, А. И. Метлинская, Н. Б. Моисеенко, Ф. Н. Мурадова, Р. Г. Мурадян, Ш. Н. Мусаелян, Е. С. Некаева, Н. М. Никитина, С. Е. Нифонтов, Е. Ю. Оболенцева, А. А. Обухова, Б. Б. Огурлиева, А. А. Одегова, Ю. В. Омарова, Н. А. Омурзакова, Ш. О. Оспанова, В. А. Павлова, Е. В. Пахомова, Л. Д. Петров, С. С. Пластинина, Д. А. Платонов, В. А. Погребецкая, Д. В. Поляков, Д. С. Поляков, Е. В. Пономаренко, Л. Л. Попова, А. А. Потанин, Н. А. Прокофьева, Ю. Д. Рабик, Н. А. Раков, А. Н. Рахимов, Н. А. Розанова, И. В. Самусь, С. Серикболкызы, Я. А. Сидоркина, А. А. Симонов, В. В. Скачкова, Р. Д. Скворцова, Д. С. Скуридин, Д. В. Соловьева, И. А. Соловьева, И. М. Сухомлинова, А. Г. Сушилова, Д. Р. Тагаева, Ю. В. Титойкина, Е. П. Тихонова, Д. С. Токмин, А. А. Толмачева, М. С. Торгунакова, К. В. Треногина, Н. А. Тростянецкая, Д. А. Трофимов, М. А. Трубникова, А. А. Туличев, А. Т. Турсунова, Н. Д. Уланова, О. В. Фатенков, О. В. Федоришина, Т. С. Филь, И. Ю. Фомина, И. С. Фоминова, И. А. Фролова, С. М. Цвингер, В. В. Цома, М. Б. Чолпонбаева, Т. И. Чудиновских, И. В. Шаврин, О. А. Шевченко, Д. Р. Шихалиев, Е. А. Шишкина, К. Ю. Шишков, С. Ю. Щербаков, Г. В. Щербакова, Е. А. Яушева

**Affiliations:** Ассоциация «Евразийская Ассоциация Терапевтов»; Национальный институт здравоохранения им. академика С. Авдалбекяна; Ассоциация «Евразийская Ассоциация Терапевтов»; Приволжский исследовательский медицинский университет; Национальный медицинский центр исследования эндокринологии; Ассоциация «Евразийская Ассоциация Терапевтов»; Российский национальный исследовательский медицинский университет им. Н.И. Пирогова; Ассоциация «Евразийская Ассоциация Терапевтов»; Северо-Западный государственный медицинский университет имени И.И. Мечникова; Ассоциация «Евразийская Ассоциация Терапевтов»; Российский национальный исследовательский медицинский университет им. Н.И. Пирогова; Первый Московский государственный медицинский университет имени И.М. Сеченова (Сеченовский Университет; Национальный медицинский исследовательский центр им. В.А. Алмазова; Волгоградский государственный медицинский университет; Саратовский государственный медицинский университет им. В.И. Разумовского; Национальный медицинский исследовательский центр кардиологии им. академика Е.И. Чазова; Ростовский государственный медицинский университет; Медицинский центр Эребуни, Клиника кардиологии и кардиохирургии; Государственный университет медицины и фармакологии им. Николая Тестемицану; Северо-Западный государственный медицинский университет имени И.И. Мечникова; Северо-Западный государственный медицинский университет имени И.И. Мечникова; Национальный исследовательский Мордовский государственный университет им. Н.П. Огарева; Приволжский исследовательский медицинский университет; Приволжский исследовательский медицинский университет; Первый Санкт-Петербургский государственный медицинский университет им. академика И.П. Павлова; Приволжский исследовательский медицинский университет; Межрегиональный клинико-диагностический центр; Казанский государственный медицинский университет; Северо-Западный государственный медицинский университет имени И.И. Мечникова; Национальный исследовательский Нижегородский государственный университет им. Н.И. Лобачевского; Самарский государственный медицинский университет; Красноярский государственный медицинский университет им. профессора В.Ф. Войно-Ясенецкого; Краевая клиническая больница; Красногорская городская больница №1; Кировская клиническая межрайонная больница; Городская клиническая больница №51 Департамента здравоохранения города Москвы; Республиканский специализированный научно-практический медицинский центр терапии и медицинской реабилитации; Городская клиническая больница №7; Петрозаводский государственный университет; Первый Санкт-Петербургский государственный медицинский университет им. академика И.П. Павлова; Читинская государственная медицинская академия; Приволжский исследовательский медицинский университет; Кировский государственный медицинский университет; Волгоградский государственный медицинский университет; Красноярский государственный медицинский университет им. профессора В.Ф. Войно-Ясенецкого; Приволжский исследовательский медицинский университет; Городская клиническая больница №13 Автозаводского района; Российская медицинская академия непрерывного профессионального образования; Российский национальный исследовательский медицинский университет им. Н.И. Пирогова; Многопрофильный Клинический Центр «Коммунарка» Департамента здравоохранения города Москвы; Гомельский государственный медицинский университет; Северо-Западный государственный медицинский университет имени И.И. Мечникова; Национальный центр кардиологии и терапии им. академика М.М. Миррахимова при Министерстве здравоохранения Кыргызской Республики, Бишкек; Национальный исследовательский Мордовский государственный университет им. Н.П. Огарева; Казахский национальный медицинский университет им. С.Д. Асфендиярова; Приволжский исследовательский медицинский университет; Пермский государственный медицинский университет им. академика Е.А. Вагнера; Северо-Западный государственный медицинский университет имени И.И. Мечникова; Южно-Уральский государственный медицинский университет; Кировская клиническая межрайонная больница; Казахский медицинский университет непрерывного образования; Городская клиническая инфекционная больница им. И. Жекеновой; Ереванский государственный медицинский университет им. Мхитара Гераци; Медицинский центр Эребуни, Клиника кардиологии и кардиохирургии; Медицинский центр Эребуни, Клиника кардиологии и кардиохирургии; Казахский национальный медицинский университет им. С.Д. Асфендиярова; Национальный центр кардиологии и терапии им. академика М.М. Миррахимова при Министерстве здравоохранения Кыргызской Республики; Национальный центр кардиологии и терапии им. академика М.М. Миррахимова при Министерстве здравоохранения Кыргызской Республики; Северо-Западный государственный медицинский университет имени И.И. Мечникова; Центральная клиническая больница с поликлиникой Управления делами Президента Российской Федерации; Саратовский государственный медицинский университет им. В.И. Разумовского; Клинический кардиологический диспансер; Приволжский исследовательский медицинский университет; Приволжский окружной медицинский центр; Петрозаводский государственный университет; Российский национальный исследовательский медицинский университет им. Н.И. Пирогова; Северо-Западный государственный медицинский университет имени И.И. Мечникова; Северо-Западный государственный медицинский университет имени И.И. Мечникова; Клинический кардиологический диспансер; Северо-Западный государственный медицинский университет имени И.И. Мечникова; Городская клиническая больница №51 Департамента здравоохранения города Москвы; Муниципальная клиническая больница №1; Пермский государственный медицинский университет им. академика Е.А. Вагнера; Поликлиника №1 Управления делами Президента Российской Федерации; Петрозаводский государственный университет; Петрозаводский государственный университет; Краевая клиническая больница; Национальный исследовательский Нижегородский государственный университет им. Н.И. Лобачевского; Северо-Западный государственный медицинский университет имени И.И. Мечникова; Ордена «Знак Почета» Пермская краевая клиническая больница; Приволжский исследовательский медицинский университет; Южно-Уральский государственный медицинский университет; Первый Московский государственный медицинский университет имени И.М. Сеченова (Сеченовский Университет); Национальный исследовательский Мордовский государственный университет им. Н.П. Огарева; Саратовский государственный медицинский университет им. В.И. Разумовского; Самарский государственный медицинский университет; Казахский национальный медицинский университет им. С.Д. Асфендиярова; Самарский государственный медицинский университет; Чапаевская центральная городская больница; Национальный центр кардиологии и терапии им. академика М.М. Миррахимова при Министерстве здравоохранения Кыргызской Республики; Северо-Западный государственный медицинский университет имени И.И. Мечникова; Республиканская больница им. В.А. Баранова; Ассоциация «Евразийская Ассоциация Терапевтов»; Общество с ограниченной ответственностью «ЭМПАТИЯ»; Казахский национальный медицинский университет им. С.Д. Асфендиярова; Приволжский окружной медицинский центр; Городская клиническая больница №25; Госпиталь для ветеранов войн; Приволжский исследовательский медицинский университет; Кировская клиническая межрайонная больница; Кировский государственный медицинский университет; Клиническая больница «РЖД-Медицина»; Красноярский государственный медицинский университет им. профессора В.Ф. Войно-Ясенецкого; Приволжский окружной медицинский центр; Читинская государственная медицинская академия; Ордена «Знак Почета» областная клиническая больница; Саратовский государственный медицинский университет им. В.И. Разумовского; Северо-Западный государственный медицинский университет имени И.И. Мечникова; Красноярский государственный медицинский университет им. профессора В.Ф. Войно-Ясенецкого; Красноярский государственный медицинский университет им. профессора В.Ф. Войно-Ясенецкого; Национальный центр кардиологии и терапии им. академика М.М. Миррахимова при Министерстве здравоохранения Кыргызской Республики; Городская клиническая больница №7; Городская клиническая больница №40 Автозаводского района; Самарский государственный медицинский университет; Российский национальный исследовательский медицинский университет им. Н.И. Пирогова; Городская поликлиника №134 Департамента здравоохранения города Москвы; Приволжский исследовательский медицинский университет; Городская клиническая больница №51 Департамента здравоохранения города Москвы; Северо-Западный государственный медицинский университет имени И.И. Мечникова; Ордена Трудового Красного Знамени Городская Клиническая Больница №1; Южно-Уральский государственный медицинский университет; Ордена Трудового Красного Знамени Городская Клиническая Больница № 1; Самарский государственный медицинский университет; Северо-Западный государственный медицинский университет имени И.И. Мечникова; Самарский государственный медицинский университет; Самарский государственный медицинский университет; Приволжский исследовательский медицинский университет; Национальный исследовательский Нижегородский государственный университет им. Н.И. Лобачевского; Городская клиническая больница №5 Нижегородского района города; Красноярский государственный медицинский университет им. профессора В.Ф. Войно-Ясенецкого; Краевая клиническая больница; Ассоциация «Евразийская Ассоциация Терапевтов»; Южно-Уральский государственный медицинский университет; Красноярский государственный медицинский университет им. профессора В.Ф. Войно-Ясенецкого; Городская клиническая больница №13 Автозаводского района; Национальный центр кардиологии и терапии им. академика М.М. Миррахимова при Министерстве здравоохранения Кыргызской Республики; Петрозаводский государственный университет; Городецкая центральная районная больница; Республиканская клиническая больница им. С.В. Каткова; Самарский государственный медицинский университет; Национальный центр кардиологии и терапии им. академика М.М. Миррахимова при Министерстве здравоохранения Кыргызской Республики; Городская поликлиника №1; Волгоградский государственный медицинский университет; Многопрофильный Клинический Центр «Коммунарка» Департамента здравоохранения города Москвы; Первый Санкт-Петербургский государственный медицинский университет им. академика И.П. Павлова; Приволжский исследовательский медицинский университет; Саратовский государственный медицинский университет им. В.И. Разумовского; Приволжский окружной медицинский центр; Гомельский государственный медицинский университет; Российская медицинская академия непрерывного профессионального образования; Национальный центр кардиологии и терапии им. академика М.М. Миррахимова при Министерстве здравоохранения Кыргызской Республики; Первый Санкт-Петербургский государственный медицинский университет им. академика И.П. Павлова; Кировская клиническая межрайонная больница; Ассоциация «Евразийская Ассоциация Терапевтов»; Северо-Западный государственный медицинский университет имени И.И. Мечникова; Первый Санкт-Петербургский государственный медицинский университет им. академика И.П. Павлова; Национальный исследовательский Нижегородский государственный университет им. Н.И. Лобачевского; Приволжский исследовательский медицинский университет; Global Medical System clinics and hospitals; Ереванский государственный медицинский университет им. Мхитара Гераци; Приволжский исследовательский медицинский университет; Саратовский государственный медицинский университет им. В.И. Разумовского; Первый Санкт-Петербургский государственный медицинский университет им. академика И.П. Павлова; Первый Санкт-Петербургский государственный медицинский университет им. академика И.П. Павлова; Первый Санкт-Петербургский государственный медицинский университет им. академика И.П. Павлова; Российский национальный исследовательский медицинский университет им. Н.И. Пирогова; Городская клиническая больница №4 Департамента здравоохранения города Москвы; Кировский государственный медицинский университет; Приволжский исследовательский медицинский университет; Национальный центр кардиологии и терапии им. академика М.М. Миррахимова при Министерстве здравоохранения Кыргызской Республики; Казахский национальный медицинский университет им. С.Д. Асфендиярова; Первый Санкт-Петербургский государственный медицинский университет им. академика И.П. Павлова; Республиканский противотуберкулезный диспансер; Центр здоровья Бричень; Приволжский исследовательский медицинский университет; Городская клиническая больница №51 Департамента здравоохранения города Москвы; Городская клиническая больница №38 Нижегородского Района; Российский национальный исследовательский медицинский университет им. Н.И. Пирогова; Приволжский исследовательский медицинский университет; Медицинский Центр «Зимамед»; Самарский государственный медицинский университет; Первый Санкт-Петербургский государственный медицинский университет им. академика И.П. Павлова; Северо-Западный государственный медицинский университет имени И.И. Мечникова; Первый Санкт-Петербургский государственный медицинский университет им. академика И.П. Павлова; Приволжский исследовательский медицинский университет; Республиканский специализированный научно-практический медицинский центр терапии и медицинской реабилитации; Красногорская городская больница №1; Кузбасская клиническая психиатрическая больница; Казахский национальный медицинский университет им. С.Д. Асфендиярова; Городская клиническая больница №51 Департамента здравоохранения города Москвы; Северо-Западный государственный медицинский университет имени И.И. Мечникова; Ордена «Знак Почета» Пермская краевая клиническая больница; Первый Санкт-Петербургский государственный медицинский университет им. академика И.П. Павлова; Первый Санкт-Петербургский государственный медицинский университет им. академика И.П. Павлова; Национальный исследовательский Нижегородский государственный университет им. Н.И. Лобачевского; Красноярский государственный медицинский университет им. профессора В.Ф. Войно-Ясенецкого; Краевая клиническая больница; Госпиталь для ветеранов войн; Северо-Западный государственный медицинский университет имени И.И. Мечникова; Республиканский специализированный научно-практический медицинский центр терапии и медицинской реабилитации; Национальный исследовательский Мордовский государственный университет им. Н.П. Огарева; Красноярский государственный медицинский университет им. профессора В.Ф. Войно-Ясенецкого; Акционерное общество «Лаборатории будущего»; Новосибирский государственный медицинский университет; Новосибирский областной клинический госпиталь ветеранов войн №3; Красноярский государственный медицинский университет им. профессора В.Ф. Войно-Ясенецкого; Ордена «Знак Почета» Пермская краевая клиническая больница; Северо-Западный государственный медицинский университет имени И.И. Мечникова; Казанский государственный медицинский университет; Городская клиническая больница №7; Ассоциация «Евразийская Ассоциация Терапевтов»; Общество с ограниченной ответственностью «Фрезениус Медиал Кеа Кубань»; Приволжский исследовательский медицинский университет; Городская клиническая больница №3; Казахский национальный медицинский университет им. С.Д. Асфендиярова; Городская клиническая больница №13 Автозаводского района; Самарский государственный медицинский университет; Российская медицинская академия непрерывного профессионального образования; Северо-Западный государственный медицинский университет имени И.И. Мечникова; Приволжский исследовательский медицинский университет; Городская поликлиника №1 Приокского района; Республиканская клиническая больница №4; Приволжский окружной медицинский центр; Читинская государственная медицинская академия; Волгоградский государственный медицинский университет; Национальный центр кардиологии и терапии им. академика М.М. Миррахимова при Министерстве здравоохранения Кыргызской Республики; Кировский государственный медицинский университет; Многофункциональный медицинский центр МЕДСИ; Самарская городская поликлиника №3; Первый Санкт-Петербургский государственный медицинский университет им. академика И.П. Павлова; Пермский государственный медицинский университет им. академика Е.А. Вагнера; Самарский государственный медицинский университет; Казанская государственная медицинская академия — филиал Российской медицинской академии непрерывного профессионального образования; Первый Московский государственный медицинский университет имени И.М. Сеченова (Сеченовский Университет; Клиническая больница «РЖД-Медицина»

**Keywords:** новая коронавирусная инфекция, SARS-CoV-2, COVID-19, ожирение, избыточная масса тела

## Abstract

ОБОСНОВАНИЕ. Имеется достаточное количество доказательств негативного влияния избыточного веса на формирование и прогрессирование патологии дыхательной системы. С учетом продолжающейся пандемии SARS-CoV-2, актуальным является определение взаимосвязей между значениями индекса массы тела (ИМТ) и особенностями клинической картины новой коронавирусной инфекции (НКИ).ЦЕЛЬ. Изучить влияние значения ИМТ на течение острой стадии НКИ и постковидного периода.МАТЕРИАЛЫ И МЕТОДЫ. АКТИВ и АКТИВ 2 - многоцентровые неинтервенционные регистры реальной клинической практики. Регистр АКТИВ состоит из амбулаторной и госпитальной непересекающихся ветвей с 6 визитами. В регистре АКТИВ 2 учитывались данные госпитализированных пациентов (3 визита). Всего в исследование были включены 6396 пациентов из регистра АКТИВ и 2968 пациентов — из регистра АКТИВ 2. Все субъекты были разделены на следующие группы: пациенты, не имеющие избыточной массы тела (n=2139), пациенты с избыточной массой тела (n=2931) и пациенты с ожирением (n=2666).РЕЗУЛЬТАТЫ. Увеличение значения ИМТ у больных, находящихся на стационарном лечении, было ассоциировано с более тяжелым течением НКИ в виде формирования острого повреждения почек (p=0,018), развития «цитокинового шторма» (p<0,001), увеличения уровня С-реактивного белка в сыворотке крови более 100 мг/л (p<0,001) и потребности в проведении таргетной терапии (p<0,001). Наличие ожирения увеличивало шансы развития миокардита в 1,84 раза (95% доверительный интервал (ДИ) 1,13–3,00) и потребности в антицитокиновой терапии в 1,7 раза (95% ДИ 1,30–2,30). У больных с ожирением 1 и 2 степеней, проходивших лечение в стационаре, наблюдалась тенденция к увеличению вероятности летального исхода, в то время как при наличии морбидного ожирения данная связь была наиболее значима (ОШ=1,78; 95% ДИ 1,13–2,70). Наряду с этим пациенты, у которых после реконвалесценции дебютировали хронические заболевания, а также присутствовали определенные жалобы, отсутствующие до инфицирования SARS-CoV-2, чаще имели ИМТ более 30 кг/м2 (p<0,001). Более того, у лиц с ожирением в возрасте старше 60 лет в 2,23 раза (95% ДИ 1,05-4,72) увеличивался шанс летального исхода в течение 3 мес после реконвалесценции.ЗАКЛЮЧЕНИЕ. Наличие избыточной массы тела и/или ожирения является значимым фактором риска тяжелого течения НКИ, поражения органов сердечно-сосудистой системы и почек. У лиц с избыточной массой тела, ожирением 1 и 2 степеней наблюдается тенденция к повышению шанса летального исхода как в острой стадии, так и в постковидном периоде, в то время как для морбидного ожирения данные связи статистически значимы. Нормализация массы тела является стратегической задачей современной медицины и может играть важную роль в профилактике патологии органов дыхания, неблагоприятного течения и осложнений НКИ.

## Abstract (en)

BACKGROUND. There is enough evidence of the negative impact of excess weight on the formation and progression of res piratory pathology. Given the continuing SARS-CoV-2 pandemic, it is relevant to determine the relationship between body mass index (BMI) and the clinical features of the novel coronavirus infection (NCI).AIM. To study the effect of BMI on the course of the acute SARS-COV-2 infection and the post-covid period.MATERIALS AND METHODS. AKTIV and AKTIV 2 are multicenter non-interventional real-world registers. The АКТИВ registry (n=6396) includes non-overlapping outpatient and inpatient arms with 6 visits in each. The АКТИВ 2 registry (n=2968) collected  the  data  of  hospitalized  patients  and  included  3  visits.  All  subjects  were  divided  into  3  groups:  not  overweight  (n=2139), overweight (n=2931) and obese (n=2666).RESULTS. A higher BMI was significantly associated with a more severe course of the infection in the form of acute kidney injury (p=0.018), cytokine storm (p<0.001), serum C-reactive protein over 100 mg/l (p<0.001), and the need for targeted therapy (p<0.001) in the hospitalized patients. Obesity increased the odds of myocarditis by 1,84 times (95% confidence interval [CI]: 1,13–3,00) and the need for anticytokine therapy by 1,7 times (95% CI: 1,30–2,30).The  patients  with  the  1st  and  2nd  degree  obesity,  undergoing  the  inpatient  treatment,  tended  to  have  a  higher  probability  of  a  mortality  rate.  While  in  case  of  morbid  obesity  patients  this  tendency  is  the  most  significant  (odds  ratio  —  1,78; 95% CI: 1,13-2,70). At the same time, the patients whose chronical diseases first appeared after the convalescence period, and those who had certain complaints missing before SARS-CoV-2 infection, more often had BMI of more than 30 kg/m2 (p<0,001).Additionally, the odds of death increased by 2,23 times (95% CI: 1,05-4,72) within 3 months after recovery in obese people over the age of 60 yearsCONCLUSION.  Overweight  and/or  obesity  is  a  significant  risk  factor  for severe  course  of  the  new  coronavirus  infection  and  the associated cardiovascular and kidney damage Overweight people and patients with the 1st and 2nd degree obesity tend to have a high risk of death of SARS-CoV-2 infection in both acute and post-covid periods. On top of that, in case of morbid obesity patients this tendency is statistically significant. Normalization of body weight is a strategic objective of modern medicine and can contribute to prevention of respiratory conditions, severe course and complications of the new coronavirus infection.

## ОБОСНОВАНИЕ

Ожирение — хроническое заболевание, определяющееся избыточным накоплением жировой ткани в организме. Данное состояние представляет угрозу для здоровья населения и является значимым фактором риска (ФР) ряда патологий внутренних органов [1]. По данным «Федеральной службы государственной статистики» (Выборочное наблюдение рациона питания населения, 2018 г.), в Российской Федерации распространенность ожирения составляет 24,5% среди женщин и 17,8% среди лиц мужского пола [2]. Установлено, что избыточный вес имеет важное значение в развитии сахарного диабета (СД) 2 типа, сердечно-сосудистых заболеваний, онкологии, остеоартрита и патологии органов дыхания (бронхиальной астмы (БА), синдрома обструктивного апноэ сна, синдрома гиповентиляции, легочной гипертензии), а также оказывает неблагоприятное влияние на качество жизни, повышает риск инвалидизации и смертности населения [3]. Данный факт подтверждается наблюдаемым увеличением заболеваемости, а также возникновением и прогрессированием клинических проявлений многих респираторных заболеваний на фоне растущего бремени ожирения. Более того, ожирение повышает восприимчивость к респираторным инфекциям и предрасполагает к более тяжелому течению инфекционного процесса, в связи с чем уровень потребности в госпитализации у таких больных выше по сравнению с лицами с нормальной массой тела [4].

Во всем мире продолжается пандемия COVID-19, которая является серьезным вызовом для системы здравоохранения. Большим количеством исследований показано, что тяжесть течения и уровень смертности от новой коронавирусной инфекции (НКИ) увеличиваются при наличии у пациентов хронических заболеваний [5–7]. С другой стороны, в период соблюдения самоизоляции из-за вынужденных ограничительных мер, нарушенного режима работы, отсутствия адекватной физической нагрузки и увеличения уровня стресса население подвергается повышенному риску ожирения [8]. Данные литературы связывают увеличение значения индекса массы тела (ИМТ) с более тяжелым течением инфекции, вызванной SARS-CoV-2, и приростом смертности как в острый период заболевания, так и в постковидном периоде [6][7][9–11].

С учетом наличия доказанных взаимосвязей между патологией дыхательной системы и ожирением, а также продолжающейся пандемии SARS-CoV-2, изучение влияния избыточного веса на течение и исходы НКИ является актуальной задачей. В данной работе представлен субанализ фрагмента общей когорты регистров АКТИВ и АКТИВ 2 на госпитальном и постгоспитальном этапах.

## ЦЕЛЬ ИССЛЕДОВАНИЯ

Изучить влияние значения ИМТ на течение острой стадии НКИ и постковидного периода.

## МАТЕРИАЛЫ И МЕТОДЫ

АКТИВ и АКТИВ 2 — многоцентровые неинтервенционные регистры реальной клинической практики, которые включали в себя пациентов, перенесших COVID-19 в период с 29.06.2020 г. по 29.11.2020 г. (АКТИВ) и с 01.10.2020 г. по 30.03.2021 г. (АКТИВ 2). Регистр АКТИВ состоит из двух непересекающихся ветвей (амбулаторная и госпитальная), в которых было предусмотрено 6 визитов: включение, на 7–12-е сутки, исход (выписка/госпитализация/смерть и т.д.) и спустя 3, 6 и 12 мес после выписки из стационара (телефонные звонки). В регистре АКТИВ 2 учитывались данные только госпитализированных пациентов и было предусмотрено 3 визита: включение, на 7–12-е сутки, исход (выписка/госпитализация/смерть и т.д.).

Дизайн регистра, подробное обоснование и методы статистического анализа, используемые для проведения данного исследования, представлены в предыдущих публикациях [12][13]. Нозологический диагноз устанавливался на основании критериев международной классификации болезней 10 пересмотра. Значения ИМТ 25–29,9 кг/м2 расценивались как избыточная масса тела. Ожирению 1-й степени соответствовал ИМТ 30–34,9 кг/м2; 2-й степени — 35–39,9 кг/м2; 3-й степени — более 40 кг/м2. Всего в субанализ были включены 6396 пациентов из регистра АКТИВ и 2968 пациентов — из регистра АКТИВ 2. Все субъекты субанализа были разделены на следующие группы: не имеющие избыточной массы тела, n=2139 (22,8%); с избыточной массой тела, n=2931 (31,3%) и с ожирением, n=2666 (28,4%), среди которых ожирение 1-й степени было зафиксировано у 1701 (63,9%) пациента, 2-й степени — у 669 (25,0%), 3-й степени — у 296 (11,1%). У остальных лиц данные об ИМТ не были внесены исследователями или внесены некорректно, так как эти параметры вводились в индивидуальной регистрационной карте по принципу «если известно». Средний возраст общей когорты составил 60 лет (Q1=49; Q3=69): в группе с нормальной массой тела — 57 лет (Q1=41; Q3=70), с избыточной массой тела — 61 год (Q1=50; Q3=69), с ожирением — 61 год (Q1=53; Q3=69), pобщ<0,001. На долю умерших приходилось 5,7% пациентов из общей когорты, 6,3% — среди лиц без избыточной массы тела, 4,4% — с избыточной массой тела и 6,7% — с ожирением, робщ<0,001. Артериальная гипертензия (АГ), фибрилляция предсердий (ФП), ишемическая болезнь сердца (ИБС), хроническая сердечная недостаточность (ХСН) и хроническая болезнь почек (ХБП) чаще встречались у больных с ожирением. В группе пациентов с избыточной массой отмечалась наибольшая распространенность СД 2 типа, в то время как у лиц с ИМТ менее 25 кг/м2 чаще встречались онкологические заболевания и анемии. При сравнительном анализе исследуемых групп по наличию хронических заболеваний дыхательной системы не было выявлено статистически значимых различий (табл. 1).

**Table:** Таблица 1. Характеристика пациентов, включенных в регистрыTable 1. Characteristics of patients included in the registers Примечание: *различия показателей статистически значимы — p<0,05 (при попарных сравнениях статистически значимые различия между всеми 3 группами); данные представлены в виде M [Q1; Q3], n (%). АГ — артериальная гипертензия; БА — бронхиальная астма; ИБС — ишемическая болезнь сердца; КТ — компьютерная томография; ОНМК — острое нарушение мозгового кровообращения; СД — сахарный диабет; ФП — фибрилляция предсердий; ХБП — хроническая болезнь почек; ХОБЛ — хроническая обструктивная болезнь легких; ХСН — хроническая сердечная недостаточность; ЧДД — частота дыхательных движений, SpO2 — сатурация.

Показатель	Общая когорта, n=7736	Общая когорта, n=9364	Пациенты без избыточной массы тела, n=2139	Пациенты с избыточной массой тела, n=2931	Пациенты с ожирением, n=2666	Робщ
Возраст	60,0 [ 49,0; 69,0]	59,0 [ 48,0; 68,0]	57,0 [ 41,0; 70,0]	61,0 [ 50,0; 69,0]	61,0 [ 53,0; 69,0]	<0,001*
Женщины	4093 (52,9%)	4960 (53%)	1129 (52,8%)	1373 (46,8%)	1591 (59,7%)	<0,001*
Умершие	438 (5,7%)	545 (5,8%)	132 (6,3%)	128 (4,4%)	178 (6,7%)	0,001*
Стадия поражения легочной ткани по КТ — 1	2601 (33,6%)	3136 (41,9%)	787 (48,3%)	992 (42,3%)	822 (36,8%)	<0,001*
Стадия поражения легочной ткани по КТ — 2	2134 (27,6%)	2563 (34,2%)	446 (27,4%)	847 (36,1%)	841 (37,7%)
Стадия поражения легочной ткани по КТ — 3	826 (10,7%)	1005 (13,4%)	146 (9,0%)	268 (11,4%)	412 (18,5%)
Стадия поражения легочной ткани по КТ — 4	178 (2,3%)	231 (3,1%)	34 (2,1%)	70 (3,0%)	74 (3,3%)
SpO2 75–94%	1799 (23,3%)	2166 (23,1%)	403 (28,3%)	696 (35,5%)	700 (41,6%)	<0,001*
SpO2 менее 75%	46 (0,6%)	55 (0,6%)	14 (1,0%)	14 (0,7%)	18 (1,1%)
ЧДД 22–29/мин	1903 (23,3%)	2314 (25,0%)	454 (21,3%)	697 (23,9%)	752 (28,3%)	<0,001*
ЧДД более 30/мин	127 (1,6%)	178 (1,9%)	27 (1,3%)	39 (1,3%)	61 (2,3%)
Температура тела более 38,6–39,0°С	1379 (17,8%)	1634 (17,7%)	25 (1,9%)	28 (1,5%)	28 (1,7%)	0,138
Температура тела более 39,0°С	5480 (7,1%)	640 (6,9%)	6 (0,4%)	6 (0,3%)	10 (0,6%)
Курение	391 (5,1%)	475 (5,09%)	130 (6,1%)	166 (5,7%)	95 (3,6%)	<0,001*
АГ	4389 (56,7%)	5289 (56,6%)	884 (41,4%)	1605 (54,9%)	1900 (71,3%)	<0,001*
ФП	568 (7,3%)	672 (7,2%)	141 (6,6%)	203 (6,9%)	224 (8,4%)	0,033*
ИБС	1739 (22,5%)	2144 (23%)	437 (20,5%)	659 (22,6%)	643 (24,1%)	0,011*
ХСН	1369 (17,7%)	1595 (17,1%)	330 (15,5%)	482 (16,5%)	557 (20,9%)	<0,001*
ОНМК	348 (4,5%)	401 (4,29%)	97 (4,5%)	132 (4,5%)	119 (4,5%)	0,991
СД 2 типа	1332 (17,2%)	1611 (17,3%)	1081 (50,5%)	1492 (50,9%)	1272 (47,7%)	<0,001*
ХБП	625 (8,1%)	716 (7,67%)	154 (7,2%)	228 (7,8%)	243 (9,1%)	0,042*
ХОБЛ	324 (4,2%)	408 (4,3%)	105 (4,9%)	115 (3,9%)	104 (3,9%)	0,146
БА	273 (3,5%)	321 (3,44%)	65 (3,0%)	99 (3,4%)	109 (4,1%)	0,129
Онкология	453 (5,9%)	536 (5,74%)	163 (7,6%)	154 (5,3%)	136 (5,1%)	<0,001*
Анемия	1648 (21,3%)	1976 (22,7%)	558 (28,0%)	588 (21,1%)	502 (19,6%)	<0,001*

## РЕЗУЛЬТАТЫ

Течение госпитального периода. Увеличение значения ИМТ было достоверно ассоциировано с более тяжелым течением НКИ в виде формирования у пациентов острого повреждения почек (ОПП), развития «цитокинового шторма», увеличения уровня С-реактивного белка (СРБ) в сыворотке крови более 100 мг/л и потребности в проведении таргетной терапии (табл. 2).

**Table:** Таблица 2. Сравнительный анализ индекса массы тела при отсутствии или наличии показателя тяжелого течения новой коронавирусной инфекцииTable 2. Comparative analysis of body mass index in the absence or presence of an indicator of the severe course of a new coronavirus infection

Показатели	Отсутствие признака, Ме [ Q1; Q3], кг/м²	Наличие признака, Ме [ Q1; Q3], кг/м²	P
Острое повреждение почек	27,8 [ 24,8; 31,6]	29,6 [ 25,1; 33,5]	0,018
«Цитокиновый шторм»	27,5 [ 24,4; 31,2]	28,7 [ 25,6; 32,8]	<0,001
Уровень С-реактивного белка в сыворотке крови более 100 мг/л	27,7 [ 24,7; 31,7]	28,7 [ 25,5; 32,7]	<0,001
Потребность в проведении таргетной терапии	27,7 [ 24,7; 31,3]	29,7 [ 26,3; 34,2]	<0,001

Также наблюдалась тенденция к наличию более высокой температуры тела у больных в остром периоде инфекционного процесса с большим значением ИМТ. Так, фебрильная лихорадка более 38,5°С чаще регистрировалась у лиц с ИМТ выше 28 кг/м² (рис. 1).

**Figure:**
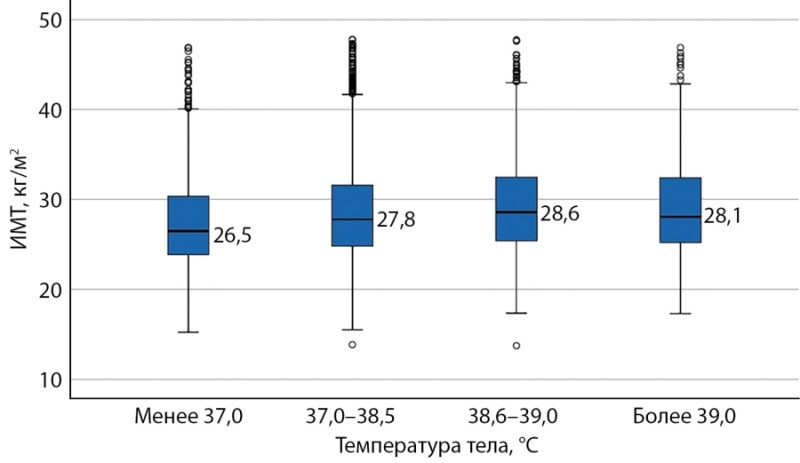
Рисунок 1. Показатель ИМТ у больных COVID-19 с различной температурой тела в остром инфекционном периоде. Примечание: ИМТ — индекс массы тела.Figure 1. BMI index in patients with COVID-19 with different body temperature in the acute infectious period. Note: BMI is body mass index.

В ходе исследования было выявлено, что ИМТ у больных НКИ, имеющих среди сопутствующих заболеваний ХБП, был достоверно выше, чем у пациентов, не имеющих патологии почек (28,6 кг/м² против 27,7 кг/м² соответственно, р=0,001) (рис. 2). При этом у пациентов, имеющих ХБП, наблюдалась тенденция к увеличению средних показателей ИМТ со стадии 1 (расчетная скорость клубочковой фильтрации (рСКФ) более 90 мл/мин/1,73 м2) до стадии 3Б (рСКФ 30–44 мл/мин/1,73 м2). Интересно, что с дальнейшим нарастанием тяжести патологии почек наблюдалось постепенное снижение показателей ИМТ (табл. 3). >

**Figure:**
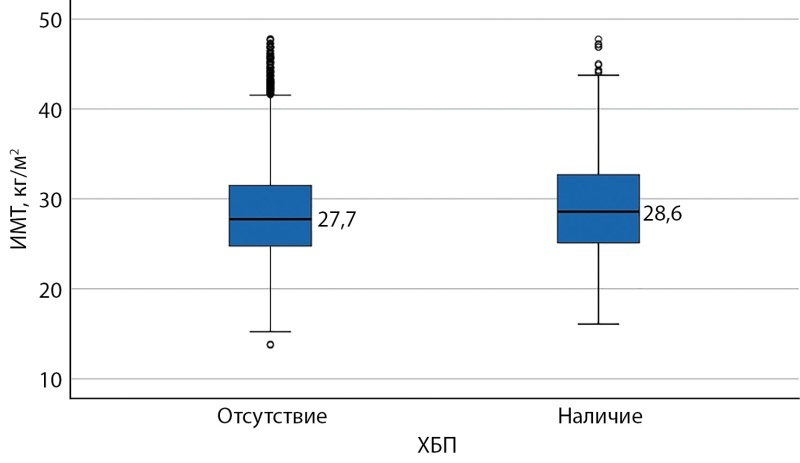
Рисунок 2. Показатель ИМТ у пациентов с новой коронавирусной инфекцией в зависимости от наличия ХБП в анамнезе.Примечание: ИМТ — индекс массы тела; ХБП — хроническая болезнь почек.Figure 2. BMI in patients with a new coronavirus infection, depending on the presence of CKD in history. Note: BMI, body mass index; CKD — chronic kidney disease

**Table:** Таблица 3. Значения индекса массы тела у пациентов с хронической болезнью почек и новой коронавирусной инфекциейTable 3. Body mass index values in patients with chronic kidney disease and NKI Примечание. рСКФ — расчетная скорость клубочковой фильтрации.

Стадия хронической болезни почек	Индекс массы тела, Ме [ Q1; Q3], кг/м²	P
Стадия 1 (рСКФ более 90 мл/мин/1,73 м2)	27,7 [ 24,7; 31,3]	Робщ<0,001 Р5 1=0,17 Р5 2=0,02 Р5 4=0,07 Р5 3Б=0,02 Р5 3А=0,008 Р1 2<0,001 Р1 4=0,25 Р1 3Б=0,02 Р1 3А<0,001 Р2 4=0,94 Р2 3Б=0,46 Р4 3Б=0,72 Р4 3А=0,67 Р3Б 3А=0,96
Стадия 2 (рСКФ 60–89 мл/мин/1,73 м2)	28,3 [ 25,1; 32,0]
Стадия 3А (рСКФ 45–59 мл/мин/1,73 м2)	28,5 [ 25,2; 33,1]
Стадия 3Б (рСКФ 30–44 мл/мин/1,73 м2)	28,8 [ 25,1; 33,3]
Стадия 4 (рСКФ 15–29 мл/мин/1,73 м2)	28,7 [ 25,0; 33,0]
Стадия 5 (рСКФ менее 15 мл/мин/1,73 м2)	26,9 [ 24,8; 29,7]

У больных COVID-19 ожирение часто сочеталось с наличием миокардита (49,2% (n=32) против 34,5% (n=2611) пациентов с ожирением без его развития, р=0,013). Кроме того, ИМТ≥30 кг/м² увеличивал шансы формирования миокардита в 1,84 раза (95% доверительный интервал (ДИ) 1,13–3,00). Среди пациентов, которым проводилась таргетная терапия, 47,3% (n=113) имели ожирение, в то время как среди пациентов без антицитокиновой терапии доля пациентов с ИМТ более 30 кг/м² составила 34,0% (n=2553), р<0,001. Таким образом, шансы получения пациентами с ожирением антицитокиновой терапии для лечения НКИ увеличивались в 1,7 раза (95% ДИ 1,30–2,30).

Наличие ожирения повышало летальность среди больных, находящихся на стационарном лечении COVID-19.

При морбидном ожирении шанс летального исхода во время острого периода НКИ увеличивался в 1,78 раза (95% ДИ 1,13–2,70) (рис. 3). При избыточной массе тела (отношение шансов (ОШ) 1,3; 95% ДИ 0,60–1,00), ожирении 1-й степени (ОШ=1,04; 95% ДИ 0,80–1,40), ожирении 2-й степени (ОШ=1,38; 95% ДИ 0,98–1,90) наблюдалась прямая зависимость увеличения шанса смерти от значения ИМТ при отсутствии статистической значимости.

**Figure:**
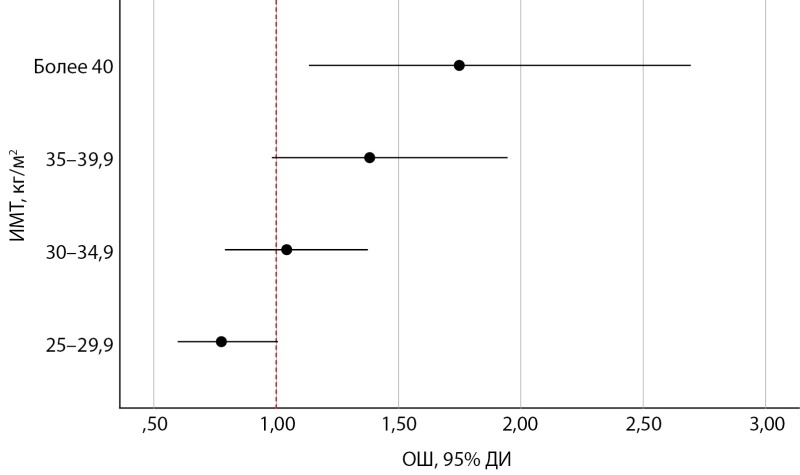
Рисунок 3. Шанс летального исхода у пациентов с НКИ в зависимости от показателей ИМТ в остром инфекционном периоде SARS-CoV-2.Примечание: ИМТ — индекс массы тела; ОШ — отношение шансов; ДИ — доверительный интервал.Figure 3. The chance of death in patients with NCI depending on BMI in the acute infectious period of SARS-CoV-2.

Течение постковидного периода. После перенесенной инфекции SARS-CoV-2 у части пациентов отмечались жалобы на общую слабость, одышку, повышение артериального давления, чувство сердцебиения, кашель, болевой синдром в области грудной клетки, потерю обоняния и/или вкуса, миалгии, диарею, артралгии, отеки нижних конечностей. Было определено, что ИМТ более 30 кг/м² был ассоциирован с увеличением шансов наличия на момент обзвона вышеперечисленных жалоб в 1,69 раза через 3 мес (95% ДИ 1,41–2,02), в 1,40 раза (95% ДИ 1,15–1,70) через 6 мес и в 1,43 раза (95% ДИ 1,14–1,80) через 12 мес после реконвалесценции по COVID-19 (p<0,001 для всех временных интервалов) (табл. 4).

**Table:** Таблица 4. Взаимосвязи наличия/отсутствия ожирения с наличием/отсутствием жалоб в постковидном периодеTable 4. Relationships between the presence/absence of obesity and the presence/absence of complaints in the post-COVID period Примечание. ИМТ — индекс массы тела; ОШ — отношение шансов; ДИ — доверительный интервал.

Период наблюдения	Показатель	Отсутствие жалоб, абс. (%)	Наличие жалоб, абс. (%)	р(общ)	ОШ (95% ДИ)	р(ОШ)
3 мес	ИМТ<30 кг/м²	759 (75,1)	916 (64,1)	<0,001	1	<0,001
ИМТ>30 кг/м²	252 (24,9)	513 (35,9)	1,69 (1,41–2,02)
6 мес	ИМТ<30 кг/м²	654 (72,5)	681 (65,3)	<0,001	1	<0,001
ИМТ>30 кг/м²	248 (27,5)	382 (34,7)	1,40 (1,15–1,70)
12 мес	ИМТ<30 кг/м²	575 (71,5)	361 (63,7)	<0,001	1	<0,001
ИМТ>30 кг/м²	229 (28,5)	206 (36,3)	1,43 (1,14–1,80)

Пациенты, у которых в течение 6 мес после реконвалесценции дебютировали хронические заболевания, отсутствующие до инфицирования SARS-CoV-2 (АГ, СД 1 и 2 типов, ИБС, ФП, БА, ХСН, острый инфаркт миокарда (ИМ), ХБП, острое нарушение мозгового кровообращения, артрит, онкологическое заболевание), чаще имели ожирение (p<0,001) (табл. 5).

**Table:** Таблица 5. Сравнительный анализ пациентов с «новыми заболеваниями» и без них, наблюдение 6 мес после реконвалесценции по COVID-19 (n=2256)Table 5. Comparative analysis of patients with and without "new diseases", follow-up 6 months after convalescence for COVID-19 (n=2256)

Параметр	Пациенты без «новых» заболеваний, n=1959	Пациенты с «новыми» заболеваниями, n=297	U-test t-test p-value
Мужчины, %	44,97	42,42	0,410
Возраст, годы, М±σ	54,4±14,75	56,14±11,26	0,050
Ожирение, %	26,39	36,7	<0,001
Ожирение <60 лет, %	14,8	25,34	<0,001

При анализе умерших и выживших пациентов в постковидном периоде было выявлено, что возрастной фактор у лиц с избыточным весом играл важную роль. Так у пациентов старше 60 лет наличие ожирения увеличивало шанс летального исхода в2,23 раза (95% ДИ 1,05–4,72), в то время как для больных с ИМТ более 30 кг/м² младше 60 лет риск неблагоприятного исхода не увеличивался. Интересно, что пациенты с резко сниженным ИМТ<18,5 кг/м2 погибали гораздо чаще, что требует дополнительного изучения (табл. 6).

**Table:** Таблица 6. Сравнительный анализ умерших и выживших пациентов в течение 3 мес после реконвалесценции по SARS-CoV-2Table 6. Comparative analysis of deceased and surviving patients within 3 months after SARS-CoV-2 convalescence Примечание. ИМТ — индекс массы тела; ДИ — доверительный интервал; ОШ — отношение шансов.

Параметр	Умершие пациенты, n=41	Выжившие пациенты, n=2144	р	ОШ (95% ДИ)
Мужчины, %	36,5	45,2	0,272	0,70 (0,36–1,32)
Ожирение, ИМТ≥30 кг/м², %	24,3	27,8	0,629	0,83 (0,40–1,72)
Ожирение ≥60 лет, %	21,9	11,2	0,032	2,23 (1,05–4,72)
Ожирение <60 лет, %	2,4	16,5	0,015	0,12 (0,01–0,91)
ИМТ<18,5 кг/м², %	8,3	0,5	<0,001	17,03 (3,47–83,41)
ИМТ≥40 кг/м², %	4,1	3,1	0,772	1,34 (0,17–10,16)

## ОБСУЖДЕНИЕ

Результаты многих исследований демонстрируют увеличение частоты встречаемости ожирения у пациентов, инфицированных SARS-CoV-2: по данным регистра в США — 41,7% больных [14], в испанском регистре — 21,2% больных [15], в российском регистре ТАРГЕТ-ВИП — 35,2% больных [16]. По результатам метаанализа 54 исследований распространенность ожирения у лиц с COVID-19 составляла 33% [17]. В более ранних публикациях, освещающих анализ регистра АКТИВ, распространенность ожирения была оценена в 35,5% пациентов [6][18]. При этом у больных, которым потребовалась госпитализация в стационар, ожирение встречалось чаще, чем у пациентов, проходящих амбулаторное лечение (38,1% против 24,8% соответственно, p<0,001). Более того, ожирение в сочетании с АГ (ОШ=1,66; 95% ДИ 1,26–2,17; p<0,001), с АГ и СД (ОШ=2,17; 95% ДИ 1,53–3,08; p<0,001), с АГ и ИБС (ОШ=2,42; 95% ДИ 1,68–3,48; p<0,001), с АГ, ИБС и ХСН (ОШ=3,86; 95% ДИ 2,57–5,80; p<0,001) приводило к увеличению вероятности летального исхода в остром периоде НКИ на госпитальном этапе [6].

В настоящем субанализе было установлено, что избыточная масса тела и ожирение ухудшали течение НКИ. Фебрильная лихорадка более 38,5°С, ОПП, «цитокиновый шторм», значимое увеличение уровня СРБ в сыворотке крови и потребность в проведении таргетной терапии наблюдались у пациентов с большим ИМТ, соответствующим избыточной массе тела. Более того, выявлено, что морбидное ожирение значимо повышало шанс летального исхода (ОШ=1,78; 95% ДИ 1,13–2,70) во время острого инфекционного периода и в постковидном периоде у пациентов старше 60 лет (ОШ=2,23; 95% ДИ 1,05-4,72). Наблюдения во всем мире показали, что 70–90% пациентов с НКИ, поступивших в отделение интенсивной терапии по поводу дыхательной недостаточности, имеют избыточный вес [9][18]. Существуют данные, которые подтверждают увеличение риска госпитализаций в целом, перевода в отделение интенсивной терапии, необходимости в проведении инвазивной вентиляции легких (ИВЛ) и неблагоприятных исходов у пациентов с избыточной массой тела [19][20]. Голландское исследование показало, что 90% пациентов с дыхательной недостаточностью на фоне инфицирования SARS-CoV-2 имели ИМТ выше 25 кг/м2 и средний ИМТ 30 кг/м2. Наряду с этим было выявлено наличие взаимосвязи между тяжестью заболевания и увеличением ИМТ [21]. Simonnet A. и соавт. [9] установили, что доля пациентов с COVID-19, которым требовалось проведение ИВЛ, нарастала прямо пропорционально ИМТ. В международном многоцентровом исследовании пациентов, госпитализированных с COVID-19, избыточный вес в целом был связан с повышенной потребностью в респираторной поддержке [22]. Крупный метаанализ с участием 3 140 413 пациентов из 167 исследований продемонстрировал, что ожирение было ассоциировано с тяжелым течением НКИ (отношение рисков (ОР)=1,52; 95% ДИ 1,41–1,63; р<0,001) и смертностью (ОР=1,09; 95% ДИ 1,02–1,16; р=0,006) [23]. Lighter J. и соавт. обнаружили, что пациенты в возрасте менее 60 лет с ИМТ 30–34 кг/м2 имели повышенный шанс госпитализации в отделение неотложной помощи (ОШ=2,00; 95% ДИ 1,60–2,60; p<0,0001) и в палату интенсивной терапии (ОШ=1,80; 95% ДИ 1,20–2,70; p=0,006) по сравнению с лицами, имеющими ИМТ менее 30 кг/м2. Более того, шанс описанных событий был выше у пациентов, имеющих ИМТ не менее 35 кг/м2 (ОШ=2,20; 95% ДИ 1,70–2,90; p<0,0001 для госпитализации в отделение неотложной помощи и ОШ=3,60; 95% ДИ 2,50–5,30; p<0,0001 для перевода в палату интенсивной терапии) [24]. В метаанализе, включившем 41 исследование, было выявлено, что лица с ожирением с большей вероятностью имели положительные результаты теста на SARS-CoV-2 (ОШ=1,50; 95% ДИ 1,37–1,63), в то время как пациенты с COVID-19 и ожирением чаще нуждались в стационарном лечении (ОШ=1,54; 95% ДИ 1,33–1,78), в переводе в отделение интенсивной терапии (ОШ=1,48; 95% ДИ 1,31– 1,65) и имели большую вероятность летального исхода (ОШ=1,14; 95% ДИ 1,04–1,26) [25].

Soeroto A. и соавт. [26] сообщили, что пациенты с более высоким ИМТ подвергались повышенному риску развития неблагоприятных исходов, определяемых как смерть, перевод в отделение интенсивной терапии, развитие острого респираторного дистресс-синдрома, тяжелое течение COVID-19, потребность в госпитализации и проведении ИВЛ. Cai Z. и соавт. [27] опубликовали результаты метаанализа, в котором было показано, что пациенты с ожирением имеют более высокий риск госпитализации, перевода в отделение интенсивной терапии и проведения ИВЛ. В исследовании Hendren N. и соавт. сообщается о том, что ожирение ассоциировалось с риском внутрибольничной смерти или потребности в ИВЛ [28]. В метаанализе 54 исследований было установлено, что наличие ожирения у пациентов с НКИ являлось значительным ФР госпитализации, потребности в проведении ИВЛ, перевода в отделение интенсивной терапии и летального исхода [16]. Таким образом, результаты, полученные в настоящем исследовании, согласуются с данными литературы, которые демонстрируют взаимосвязи повышения ИМТ с более тяжелым течением острого периода НКИ и неблагоприятными исходами [29][30].

Имеется множество данных, подтверждающих неблагоприятное влияние НКИ на функцию почек, в том числе на повышение частоты ОПП у пациентов с избыточной массой тела и ожирением [31][32]. Поражение почек при НКИ является многофакторным процессом. Во-первых, SARS-CoV-2 может оказывать прямое воздействие на паренхиму органа посредством активации ангиотензинпревращающего фермента 2, который является субстратом для проникновения вируса. Во-вторых, развитие гипервоспалительной реакции способно приводить либо к прямому повреждению почек, либо к опосредованному повреждению вследствие сепсиса, шока, гипоксии и рабдомиолиза. В-третьих, микротромбозы во время НКИ являются ФР острой ишемии почек [33]. В свою очередь, ожирение сопровождается увеличением синтеза адипонектина, лептина, провоспалительных цитокинов и других функционально активных молекул, что в совокупности с инсулинорезистентностью также может служить значимым ФР развития патологии почек [34][35]. Более того, избыток жировой ткани приводит к компрессии почек и повышению внутрипочечного давления [36]. Наличие субклинической ХБП, нарушений углеводного и липидного обменов,а также ряда других сопутствующих заболеваний увеличивает риск ОПП у пациентов с ожирением [34][35]. В проведенном исследовании было выявлено, что избыточная масса тела и ожирение ассоциировались с повышением частоты развития ОПП вострый период НКИ. Также было установлено, что наличие ХБП у пациентов, госпитализированных по поводу COVID-19, было связано с более высоким ИМТ, соответствующим избыточной массе тела. Наряду с этим были определены взаимосвязи увеличения ИМТ с выраженностью ХБП с 1 по 3Б стадии. Наличие обратной зависимости с 3Б стадии заболевания требует дальнейшего детального изучения.

Острый миокардит является сложной диагностической проблемой для клиницистов. У пациентов с НКИ его встречаемость составляет 2,4 на 1000 госпитализаций, а частота повреждения сердечной мышцы (определяемое повышением уровня тропонина) варьирует от 19 до 28% [36][37]. В систематическом обзоре Haussner W. и соавт. сообщается, что около 50–58% пациентов с миокардитом имеют по крайней мере одно из сопутствующих заболеваний: АГ, СД, ожирение, БА или хроническую обструктивную болезнь легких [38]. Однако в литературе данные по развитию миокардита у пациентов с НКИ являются неоднородными в отношении демографических и клинических проявлений, в том числе по результатам инструментальных игистологических методов исследования [39]. Данные регистра АКТИВ позволяют говорить о том, что наличие миокардита во время НКИ было чаще ассоциировано с ожирением и с избыточной массой тела, чем с нормальной массой тела. Исходя из вышесказанного, результаты, полученные в ходе выполнения настоящего субанализа, согласуются с данными литературы о негативном влиянии избыточной массы тела и ожирения на поражение почек и сердечной мышцы в остром периоде SARS-CoV-2.

Актуальным вопросом представляется изучение течения последствий COVID-19 в виде постковидного периода или long-COVID. Elkan M. и соавт. сообщили о том, что пациенты после реконвалесценции по SARS-CоV-2 и выписки из стационара имели целый ряд симптомов и ощущали ухудшение состояния здоровья в течение нескольких месяцев. Наиболее часто отмечались жалобы на общую слабость и утомляемость, миалгии, одышку. При этом как минимум один симптом наблюдался у 57% пациентов, а два и более — у 34% пациентов [40]. В обзоре Nittas V. и соавт. [41] было показано, что пациенты после выздоровления чаще всего страдали от усталости, одышки, нарушений обоняния, головной боли, болевого синдрома в области грудной клетки, потери памяти и нарушений сна. В настоящем субанализе и в более ранних публикациях по данным анализа регистра АКТИВ было показано, что через 6 мес после реконвалесценции по НКИ наличие ожирения в любом возрасте (36,7% против 26,39%, р<0,001) было ассоциировано с дебютом «новых» хронических заболеваний (АГ, СД 1 и 2 типа, ИБС, ФП, артрит, инсульт, БА, онкологическое заболевание, ХСН, ИМ, ХБП). После перенесенной инфекции SARS-CoV-2 у части пациентов отмечались жалобы на общую слабость, одышку, повышение артериального давления, чувство сердцебиения, кашель, болевой синдром в области грудной клетки, потерю обоняния и/или вкуса, миалгии, диарею, артралгии, отеки нижних конечностей. ИМТ более 30 кг/м² был ассоциирован с увеличением шансов наличия на момент телефонного обзвона вышеперечисленных жалоб. В течение 3 мес постковидного периода наличие ожирения у лиц старше 60 лет приводило к увеличению шанса летального исхода (ОШ=2,23; 95% ДИ 1,05–4,72; p=0,032). Интересно, что у пациентов с ИМТ<18,5 кг/м2 также отмечалось значимое увеличение вероятности неблагоприятного исхода (ОШ=17,03; 95% ДИ 3,47–83,41; р<0,001) [10], что также требует дальнейшего изучения. В исследовании Günster  C. и соавт. [42], включавшем 8679 пациентов из Германии, было показано, что ИМТ≥40 кг/м2 являлся одним из ФР смертности от всех причин в течение 180 сут после реконвалесценции (ОШ=2,01; 95% ДИ 1,33–3,05; p<0,01). Таким образом, результаты проведенного исследования схожи с данными литературы как с точки зрения наличия клинических проявлений у пациентов после реконвалесценции по НКИ, так и роли избыточной массы тела и ожирения в их сохранении и увеличении вероятности неблагоприятного исхода.

## ОГРАНИЧЕНИЯ ИССЛЕДОВАНИЯ

АКТИВ и АКТИВ 2 являются регистрами реальной клинической практики. Данные для некоторых переменных вводились по принципу «если известно» и не были обязательны для заполнения. В связи с этим существует некоторая потеря данных на этапе их ввода врачами-исследователями, и точность информации, полученной при телефонном разговоре, может быть ограничена. Для параметров, используемых в субанализе, доля заполнения составила 68%. Количество наблюдений позволяет сделать выводы из полученных результатов. Также регистры заполнялись на разных этапах изменения федеральных клинических рекомендаций по ведению пациентов с НКИ (изменения касались в основном вопросов терапии пациентов). Необходимо учесть, что в начале пандемии (весна и лето 2020 г.) фактическое количество госпитализаций по причине инфицирования SARS-CoV-2 было выше, чем необходимое по прямым медицинским показаниям, ввиду недостаточного количества информации о данной патологии, в связи с чем можно считать, что в регистре представлены пациенты с различной степенью тяжести COVID-19.

## ЗАКЛЮЧЕНИЕ

Ожирение — патология, которая способна приводить к нарушению функции дыхания и увеличивать риск возникновения инфекционных заболеваний. Жировая ткань имеет важное значение в патогенезе инфекции, вызванной SARS-CoV-2. Наличие избыточной массы тела и/или ожирения является значимым ФР тяжелого течения НКИ, поражения органов сердечно-сосудистой системы и почек. Более того, у лиц с избыточной массой тела, ожирением 1 и 2 степеней наблюдается тенденция к повышению шанса летального исхода как в острой стадии, так и в постковидном периоде, в то время как для морбидного ожирения данные связи статистически значимы. Нормализация массы тела является стратегической задачей современной медицины и может играть важную роль в профилактике патологии органов дыхания, неблагоприятного течения и осложнений НКИ.

## ДОПОЛНИТЕЛЬНАЯ ИНФОРМАЦИЯ

Источник финансирования. Авторы декларируют отсутствие внешнего финансирования для проведения исследования и публикации статьи.

Конфликт интересов. Авторы декларируют отсутствие явных и потенциальных конфликтов интересов, связанных с публикацией настоящей статьи.

Участие авторов. Авторы декларируют соответствие своего авторства международным критериям ICMJE. Все авторы в равной степени участвовали в подготовке публикации: концепция статьи, сбор и обработка материалов, анализ полученных данных, написание, проверка и утверждение текста статьи.

Все авторы одобрили финальную версию статьи перед публикацией, выразили согласие нести ответственность за все аспекты работы, подразумевающую надлежащее изучение и решение вопросов, связанных с точностью или добросовестностью любой части работы.
